# Constructing a risk prediction model for post-TKA DVT formation based on machine learning methods

**DOI:** 10.1097/MD.0000000000046326

**Published:** 2025-12-26

**Authors:** Huanya Li, Zhicheng He, Pengcui Li, Li Guo, Xiaochun Wei

**Affiliations:** a Academy of Medical Sciences, Shanxi Medical University, Shanxi, China; b Department of Orthopedics, Second Hospital of Shanxi Medical University, Taiyuan, Shanxi, China.

**Keywords:** artificial knee replacement, deep vein thrombosis, machine learning, risk prediction model

## Abstract

To construct and compare the performance of risk prediction models for deep vein thrombosis (DVT) in the lower extremities after total knee arthroplasty (TKA) using logistic regression, XGBoost, Random Forest, AdaBoost, gradient boosting decision tree, and KNN models. The study also aims to explore the risk factors for DVT after TKA, providing a reference for evaluating and preventing DVT post-TKA. A retrospective collection of medical records from 3058 patients who underwent TKA at the Second Hospital of Shanxi Medical University from July 2020 to July 2023 was conducted. Based on inclusion and exclusion criteria, 1238 patient cases were selected for the study. Lasso regression and Boruta feature selection methods were used to identify risk factors associated with post-TKA DVT. Using R 4.2.3 statistical software, the dataset was randomly divided into training and validation sets. Six machine learning models were established, and clinical decision curves, calibration curves, and receiver operating characteristic curve curve metrics were used to evaluate model performance. The models were then compared to select the best predictive model, which was further visualized using the SHAP method. Eleven independent variables, including anemia, blood transfusion volume, blood loss, HCT, D- dimer, thrombin time (TT), CL, anesthesia duration, surgery duration, activated partial thromboplastin time (APTT), and postoperative pain scores, were identified as characteristic factors and included in the 6 machine learning models. The optimal predictive model was found to be the Logistic model. According to SHAP values, shorter APTT, longer anesthesia duration, elevated TT, higher postoperative pain scores, reduced D-dimer, decreased CL, increased blood transfusion volume, increased blood loss, shorter surgery duration, and anemia increase the risk of DVT in the lower extremities post-TKA. In constructing a model to predict the risk of deep vein thrombosis in the lower extremities after artificial knee joint replacement surgery, the Logistic model demonstrated outstanding performance and excellent generalization ability. This model can identify risk factors for deep vein thrombosis complications after total knee arthroplasty, enabling clinicians to manage patients with these risk factors more comprehensively and meticulously, thereby potentially reducing the incidence of deep vein thrombosis in the lower extremities after total knee arthroplasty.

## 1. Introduction

Total knee arthroplasty (TKA) is a common surgical treatment for knee diseases, but it carries a high risk of postoperative deep vein thrombosis (DVT).^[1]^ The occurrence of DVT not only affects the patient’s recovery process but may also lead to serious complications such as pulmonary embolism (PE). With the global aging population and changes in lifestyle, the incidence of DVT is gradually increasing, especially in patients undergoing major surgery or those who have been bedridden for a long time. Among them, TKA is one of the main surgeries leading to the occurrence of DVT.^[2]^ Therefore, studying the risk factors for DVT after TKA is of great significance for preventing and reducing the incidence of DVT. In recent years, there has been an increasing amount of research on DVT, especially regarding its risk factors, prevention methods, and treatment strategies. Many studies have focused on the risk of DVT after knee replacement, exploring the effects of age, gender, preoperative health status, medication use, and other potential risk factors on the occurrence of DVT.^[3]^ Additionally, there are studies that pay attention to the efficacy and safety of different anticoagulants in preventing DVT.

It is worth noting that, in addition to traditional clinical and biomarker risk assessment methods, artificial intelligence technology has also begun to be applied to the risk prediction of DVT. Machine learning prediction models have been widely used in the medical field, especially in the prediction of DVT formation. These models can effectively predict the risk of DVT occurrence by analyzing various clinical indicators and biomarker data of patients, providing doctors with more accurate and timely decision support, thereby helping doctors make early diagnoses and treatments, and better prevent and manage DVT.

This study collected clinical case data of patients undergoing total knee arthroplasty, and based on machine learning methods, constructed risk prediction models for the occurrence of deep vein thrombosis after total knee arthroplasty using 6 machine learning methods: LR model, XGBoost model, RF model, AdaBoost model, GBDT model, and KNN model. The performance of the models was compared, the optimal model was selected, and the SHAP method was used for interpretability analysis of the model, revealing the risk factors and impact degree of DVT occurrence after TKA, to provide a reference for the subsequent establishment of a more complete risk prediction model for the occurrence of deep vein thrombosis after total knee arthroplasty.

## 2. Objects and methods

### 2.1. Clinical data

A retrospective collection of medical records from 3058 patients who underwent TKA in the Second Hospital of Shanxi Medical University from July 2020 to July 2023 was conducted. After screening through inclusion and exclusion criteria, only 1238 patients were retained for analysis.

### 2.2. Inclusion criteria

The inclusion criteria for this study are as follows: Patients who underwent TKA in our hospital; age ≥ 18 years old; hospital stay > 3 days; the surgical method was a primary unilateral total knee arthroplasty; and complete medical records.

### 2.3. Exclusion criteria

The exclusion criteria for this study are as follows: other surgical methods such as noncompartmental replacement, knee revision surgery, and simultaneous bilateral knee replacement; preoperative lower extremity vascular ultrasound diagnosis of lower extremity DVT; preoperative CT pulmonary angiography diagnosis of PE; history of thrombosis; and concurrent hematological diseases or long-term use of anticoagulants; (6)Incomplete or missing medical records.

### 2.4. Observation index

Basic Information: Age, gender, ethnicity, body mass index, residence, occupation, primary diagnosis; past medical history: smoking history, alcohol abuse history, history of primary hypertension, diabetes history, sleep apnea syndrome history, myocardial infarction history, thyroid hyperthyroidism and hypothyroidism history, allergy history, depression, asthma history, coronary atherosclerotic heart disease history, digestive system history, neuromuscular skeletal system history, anemia history, coagulation abnormalities, hyperlipidemia, varicose veins, venous thrombosis, peripheral vascular disease; surgical indicators: name of surgery, anesthesia method, ASA classification, physical status, RCRI index, tourniquet use time, total input volume, blood transfusion volume, blood loss volume, anesthesia time, operation time, postoperative resting pain score, postoperative exercise pain score, Ramsay sedation scale, surgical risk assessment; biochemical indicators: hemoglobin (Hb), hematocrit (HCT), white blood cells (WBC), platelet count (PLTs), postoperative prothrombin time (PT), activated partial thromboplastin time (APTT), fibrinogen (FIB), D-dimer, alanine aminotransferase (ALT), aspartate aminotransferase (AST), albumin (ALB), blood glucose (GLU), potassium (K), sodium (Na), chloride (Cl), urea (Urea), creatinine (CREA), thrombin time (TT), erythrocyte sedimentation rate (ESR), C- reactive protein (CRP).

### 2.5. Statistical treatment

The Excel was used for the recording and verification of questionnaire data, while SPSS 26.0 and RStudio 4.2.3 software were utilized for statistical analysis and graphing. The measurement data are expressed as mean ± standard deviation (SD), using independent sample t-test; count data are expressed as cases (%), using χ2 test; in univariate analysis, categorical variables use χ^2^ test or Mann–Whitney *U* test, and continuous variables that follow a normal distribution use t-test. For features with a missing rate not exceeding 30%, multiple imputation (MI) is used to fill in the data. Lasso regression and Boruta methods were used to select feature variables, and the rms package in RStudio software was used to construct a risk prediction model based on machine learning methods. A grid search method with 10-fold cross- validation was used to optimize the hyperparameters of the machine learning model, evaluating the accuracy, discrimination, and clinical utility of the model. The SHAP method was used for visual explanation of the model. The test level α = 0.05.

## 3. Results

### 3.1. Comparison of baseline data between 2 groups of patients

After excluding 1820 patients who did not meet the inclusion criteria, a total of 1238 patients remained for this study. There were significant differences between the 2 groups in terms of age, BMI, anesthesia method, history of allergies, anemia, RCRI index, PT, APTT, D-dimer, GLU, Urea, TT, volume of blood transfusion, duration of anesthesia, operation time, and postoperative movement pain score (*P* < .05), as shown in Table 1.

**Table 1:** Comparison of baseline data between the 2 groups of patients.

Variable	Total (n = 1238)	Lower limb DVT		Statistics	*P*
		No (n = 984)	Yes (n = 254)		
Gender, *n* (%)				χ^2^ = 0.223*	.636
Male	356 (28.756)	286 (29.065)	70 (27.559)		
Female	882 (71.244)	698 (70.935)	184 (72.441)		
Age, mean ± SD	67.637 ± 7.169	67.408 ± 7.183	68.524 ± 7.057	*t* = -2.216†	.027
Nation, *n* (%)				_-_ ‡	1.000
Han	1234 (99.677)	980 (99.593)	254 (100.000)		
Hui	3 (0.242)	3 (0.305)	0 (0.000)		
Korean	1 (0.081)	1 (0.102)	0 (0.000)		
Length of hospital stay, M (Q1, Q_3_)	7.000 (7.000,9.000)	7.000 (7.000,9.000)	7.000 (7.000, 9.000)	*Z* = -1.797§	.072
Residence, *n* (%)				χ^2^ = 0.749*	.387
Town	566 (45.719)	456 (46.341)	110 (43.307)		
Village	672 (54.281)	528 (53.659)	144 (56.693)		
Occupation, *n* (%)				_-_ ∥	.297
Retired personnel	360 (29.079)	293 (29.776)	67 (26.378)		
Farmer	697 (56.300)	543 (55.183)	154 (60.630)		
Unemployed	107 (8.643)	84 (8.537)	23 (9.055)		
Worker	30 (2.423)	28 (2.846)	2 (0.787)		
Freelancer	20 (1.616)	17 (1.728)	3 (1.181)		
Employee	12 (0.969)	10 (1.016)	2 (0.787)		
Others	6 (0.485)	3 (0.305)	3 (1.181)		
Professional and technical personnel	4 (0.323)	4 (0.407)	0 (0.000)		
Civil servant	1 (0.081)	1 (0.102)	0 (0.000)		
Student	1 (0.081)	1 (0.102)	0 (0.000)		
Primary diagnosis, *n* (%)				_-_ ∥	.123
Osteoarthritis of the knee	1148 (92.730)	909 (92.378)	239 (94.094)		
Degenerative disease of the knee joint	45 (3.635)	39 (3.963)	6 (2.362)		
Rheumatoid arthritis	37 (2.989)	31 (3.150)	6 (2.362)		
Arthralgia	5 (0.404)	4 (0.407)	1 (0.394)		
Avascular necrosis of the femoral head	2 (0.162)	0 (0.000)	2 (0.787)		
Knee chondritis	1 (0.081)	1 (0.102)	0 (0.000)		
BMI, mean ± SD	26.112 ± 3.496	26.213 ± 3.497	25.722 ± 3.468	*t* = 1.993†	.047
Surgical procedure name, *n* (%)				χ^2^ = 1.228*	.268
Left TKA	571 (46.123)	446 (45.325)	125 (49.213)		
Right TKA	667 (53.877)	538 (54.675)	129 (50.787)		
Anesthesia method, *n* (%)				_-_ ∥	.010
Compound anesthesia	1058 (85.460)	837 (85.061)	221 (87.008)		
Subarachnoid block	102 (8.239)	92 (9.350)	10 (3.937)		
General anesthesia	48 (3.877)	33 (3.354)	15 (5.906)		
Epidural anesthesia	23 (1.858)	15 (1.524)	8 (3.150)		
Resting compound anesthesia	4 (0.323)	4 (0.407)	0 (0.000)		
Intravenous anesthesia	2 (0.162)	2 (0.203)	0 (0.000)		
Others	1 (0.081)	1 (0.102)	0 (0.000)		
History of smoking, *n* (%)				χ^2^ = 0.253*	.615
No	1146 (92.569)	909 (92.378)	237 (93.307)		
Yes	92 (7.431)	75 (7.622)	17 (6.693)		
History of drinking, *n* (%)				χ^2^ = 0.000¶	1.000
No	1219 (98.465)	969 (98.476)	250 (98.425)		
Yes	19 (1.535)	15 (1.524)	4 (1.575)		
History of diabetes, *n* (%)				χ^2^ = 1.278*	.258
No	1049 (84.733)	828 (84.146)	221 (87.008)		
Yes	189 (15.267)	156 (15.854)	33 (12.992)		
Primary hypertension, *n* (%)				χ^2^ = 0.469*	.493
No	537 (43.376)	422 (42.886)	115 (45.276)		
Yes	701 (56.624)	562 (57.114)	139 (54.724)		
OSA, *n* (%)				_-_ ‡	.587
No	1234 (99.677)	980 (99.593)	254 (100.000)		
Yes	4 (0.323)	4 (0.407)	0 (0.000)		
MI, *n* (%)				χ^2^ = 1.986¶	.159
NO	1226 (99.031)	972 (98.780)	254 (100.000)		
Yes	12 (0.969)	12 (1.220)	0 (0.000)		
Hyperthyroidism/hypothyro idism, *n* (%)				χ^2^ = 0.000¶	1.000
No	1214 (98.061)	965 (98.069)	249 (98.031)		
Yes	24 (1.939)	19 (1.931)	5 (1.969)		
Allergy history, *n* (%)				χ^2^ = 9.624*	.002
No	1109 (89.580)	868 (88.211)	241 (94.882)		
Yes	129 (10.420)	116 (11.789)	13 (5.118)		
Depression, *n* (%)				χ^2^ = 0.000¶	1.000
No	1233 (99.596)	980 (99.593)	253 (99.606)		
Yes	5 (0.404)	4 (0.407)	1 (0.394)		
Asthma, *n* (%)				χ^2^ = 0.000¶	1.000
No	1230 (99.354)	978 (99.390)	252 (99.213)		
Yes	8 (0.646)	6 (0.610)	2 (0.787)		
CAD, *n* (%)				χ^2^ = 0.492¶	.483
No	1220 (98.546)	968 (98.374)	252 (99.213)		
Yes	18 (1.454)	16 (1.626)	2 (0.787)		
Gastrointestinal disorders, *n* (%)				χ^2^ = 1.736¶	.188
No	1227 (99.111)	973 (98.882)	254 (100.000)		
Yes	11 (0.889)	11 (1.118)	0 (0.000)		
Neuromuscular skeletal system, *n* (%)				χ^2^ = 0.107*	.744
No	1000 (80.775)	793 (80.589)	207 (81.496)		
Yes	238 (19.225)	191 (19.411)	47 (18.504)		
Anemia, *n* (%)				χ^2^ = 7.056¶	.008
No	1216 (98.223)	972 (98.780)	244 (96.063)		
Yes	22 (1.777)	12 (1.220)	10 (3.937)		
Coagulation disorders, *n* (%)				χ^2^ = 0.000¶	1.000
No	1230 (99.354)	978 (99.390)	252 (99.213)		
Yes	8 (0.646)	6 (0.610)	2 (0.787)		
Hyperlipidemia, *n* (%)				_-_ ‡	.587
No	1234 (99.677)	980 (99.593)	254 (100.000)		
Yes	4 (0.323)	4 (0.407)	0 (0.000)		
Varicose veins, *n* (%)				_-_ ‡	1.000
No	1236 (99.838)	982 (99.797)	254 (100.000)		
Yes	2 (0.162)	2 (0.203)	0 (0.000)		
Thrombosis, *n* (%)				χ^2^ = 0.001¶	.978
No	1226 (99.031)	975 (99.085)	251 (98.819)		
Yes	12 (0.969)	9 (0.915)	3 (1.181)		
PVD, *n* (%)				χ^2^ = 1.884*	.170
No	1209 (97.658)	958 (97.358)	251 (98.819)		
Yes	29 (2.342)	26 (2.642)	3 (1.181)		
ASA classification, *n* (%)				*Z* = -0.238§	.812
1	27 (2.197)	24 (2.459)	3 (1.186)		
2	984 (80.065)	778 (79.713)	206 (81.423)		
3	216 (17.575)	173 (17.725)	43 (16.996)		
4	2 (0.163)	1 (0.102)	1 (0.395)		
NYHA class, *n* (%)				*Z* = -0.029§	.977
1	368 (30.744)	295 (30.890)	73 (30.165)		
2	791 (66.082)	628 (65.759)	163 (67.355)		
3	38 (3.175)	32 (3.351)	6 (2.479)		
Physical functioning, *n* (%)				*Z* = 1.489§	.136
1	1088 (91.352)	863 (90.747)	225 (93.750)		
2	65 (5.458)	55 (5.783)	10 (4.167)		
3	38 (3.191)	33 (3.470)	5 (2.083)		
RCRI, *n* (%)				*Z* = 1.995§	.046
0	1059 (85.958)	832 (84.985)	227 (89.723)		
1	132 (10.714)	110 (11.236)	22 (8.696)		
2	26 (2.110)	23 (2.349)	3 (1.186)		
3	7 (0.568)	6 (0.613)	1 (0.395)		
5	4 (0.325)	4 (0.409)	0 (0.000)		
10	1 (0.081)	1 (0.102)	0 (0.000)		
15	3 (0.244)	3 (0.306)	0 (0.000)		
Hb, mean ± SD	134.673 ± 14.220	134.809 ± 14.317	134.146 ± 13.853	*t* = 0.662†	.508
HCT, mean ± SD	0.409 ± 0.039	0.409 ± 0.039	0.409 ± 0.040	*t* = 0.020†	.984
WBC, mean ± SD	6.114 ± 1.504	6.082 ± 1.530	6.235 ± 1.397	*t* = -1.448†	.148
PLTS, mean ± SD	230.443 ± 69.471	229.608 ± 68.898	233.673 ± 71.692	*t* = -0.831†	.406
PT, M (Q1, Q_3_)	13.400 (12.700, 14.100)	13.500 (12.900, 14.200)	13.100 (11.312, 13.800)	*Z* = 6.443§	<.001
APTT, Mean ± SD	30.397 ± 3.270	30.600 ± 3.206	29.611 ± 3.399	*t* = 4.330†	<.001
FIB, M (Q1, Q_3_)	2.940 (2.600, 3.340)	2.940 (2.603, 3.340)	2.950 (2.570, 3.330)	*Z* = 0.401§	.688
D-dimer, M (Q1, Q_3_)	109.000 (59.000, 179.000)	114.000 (69.000, 185.000)	87.000 (0.420, 148.000)	*Z* = 5.476§	<.001
ALT, M (Q1, Q_3_)	16.900 (13.100, 23.300)	16.800 (13.000, 23.300)	17.150 (13.400, 23.075)	*Z* = -0.402§	.688
AST, M (Q1, Q_3_)	20.700 (17.900, 24.900)	20.800 (18.000, 24.800)	20.450 (17.825, 25.175)	*Z* = 0.269§	.788
ALB, Mean ± SD	40.471 ± 3.525	40.501 ± 3.170	40.356 ± 4.659	*t* = 0.581†	.561
GLU, M (Q1, Q_3_)	5.320 (4.853, 6.080)	5.300 (4.840, 6.010)	5.480 (4.908, 6.557)	*Z* = -2.558§	.011
K, M (Q1, Q_3_)	3.990 (3.780, 4.180)	3.990 (3.775, 4.180)	4.010 (3.790, 4.188)	*Z* = -0.593§	.553
Na, mean ± SD	139.798 ± 10.752	139.745 ± 11.601	140.002 ± 6.506	*t* = -0.340†	.734
Cl, mean ± SD	105.412 ± 8.310	105.606 ± 7.538	104.661 ± 10.775	*t* = 1.617†	.106
Urea, M (Q1, Q_3_)	5.600 (4.700, 6.600)	5.500 (4.600, 6.575)	5.800 (4.800, 6.700)	*Z* = -2.449§	.014
CREA, mean ± SD	60.878 ± 14.069	60.556 ± 14.013	62.121 ± 14.242	*t* = -1.581†	.114
TT, mean ± SD	15.265 ± 1.782	15.124 ± 1.653	15.816 ± 2.130	*t* = -5.564†	<.001
ESR, M (Q1, Q_3_)	13.000 (8.000, 22.000)	13.000 (8.000, 22.000)	13.000 (7.000, 23.000)	*Z* = -0.410§	.682
CRP, *n* (%)				χ^2^ = 1.528*	.216
Normal	1001 (83.906)	784 (83.227)	217 (86.454)		
High	192 (16.094)	158 (16.773)	34 (13.546)		
Tourniquet time, M (Q_1_, Q_3_)	70.000 (60.000, 85.000)	70.000 (60.000, 85.000)	70.000 (60.000, 85.000)	*Z* = -0.411§	.681
Total intake, mean ± SD	1378.960 ± 354.391	1370.298 ± 355.204	1412.520 ± 349.894	*t* = -1.694†	.090
Transfusion volume, M (Q_1_, Q_3_)	0.000 (0.000, 0.000)	0.000 (0.000, 0.000)	0.000 (0.000, 0.000)	*Z* = -2.843§	.004
Blood loss, M (Q1, Q_3_)	0.000 (0.000, 50.000)	0.000 (0.000, 50.000)	0.000 (0.000, 100.000)	*Z* = -1.831§	.067
Anesthetic time, mean ± SD	149.567 ± 46.953	147.018 ± 48.303	159.429 ± 39.889	*t* = -3.775†	<.001
Surgical duration, M (Q_1_, Q_3_)	92.000 (75.000, 115.000)	90.000 (74.000, 113.000)	100.000 (80.000, 122.000)	*Z* = -3.933§	<.001
Resting pain score after surgery, *n* (%)				*Z* = 1.016§	.310
0	575 (49.957)	451 (48.915)	124 (54.148)		
1	418 (36.316)	346 (37.527)	72 (31.441)		
2	149 (12.945)	120 (13.015)	29 (12.664)		
3	4 (0.348)	1 (0.108)	3 (1.310)		
4	2 (0.174)	2 (0.217)	0 (0.000)		
6	1 (0.087)	0 (0.000)	1 (0.437)		
7	2 (0.174)	2 (0.217)	0 (0.000)		
Active pain score after surgery, *n* (%)				*Z* = -2.823§	.005
0	167 (14.509)	155 (16.811)	12 (5.240)		
1	474 (41.182)	366 (39.696)	108 (47.162)		
2	387 (33.623)	310 (33.623)	77 (33.624)		
3	112 (9.731)	83 (9.002)	29 (12.664)		
4	4 (0.348)	4 (0.434)	0 (0.000)		
5	4 (0.348)	2 (0.217)	2 (0.873)		
7	1 (0.087)	0 (0.000)	1 (0.437)		
8	2 (0.174)	2 (0.217)	0 (0.000)		
Ramsay sedation scale, *n* (%)				*Z* = 0.374§	.709
0	7 (0.644)	7 (0.797)	0 (0.000)		
1	17 (1.564)	11 (1.253)	6 (2.871)		
2	1061 (97.608)	859 (97.836)	202 (96.651)		
3	2 (0.184)	1 (0.114)	1 (0.478)		
Surgical risk assessment, *n* (%)				*Z* = 0.404§	.687
0	1039 (84.816)	824 (84.600)	215 (85.657)		
1	183 (14.939)	148 (15.195)	35 (13.944)		
2	3 (0.245)	2 (0.205)	1 (0.398)		

ALB = albumin, ALT = alanine aminotransferase, APTT = activated partial thromboplastin time, AST = aspartate aminotransferase, BMI = body mass index, Cl = chloride, CREA = creatinine, CRP = reactive protein, DVT = deep vein thrombosis, ESR = erythrocyte sedimentation rate, FIB = fibrinogen, GLU = blood glucose, GTT = global thrombus test, Hb = hemoglobin, HCT = hematocrit, LT = lysis time, MI = multiple imputation, Na = sodium, OA = osteoarthritis, PE = pulmonary embolism, PLTs = platelet count, PT = postoperative prothrombin time, PT-INR = prothrombin time international normalized ratio, PVD = peripheral vascular disease, RF = Random Forest, SD = standard deviation, TKA = total knee arthroplasty, TT = thrombin time, Urea = urea, WBC = white blood cells.

* Pearson χ^2^ test.

† Independent samples t-test.

‡ Fisher exact probability method.

§ Mann–Whitney *U* test.

∥ Fisher exact probability method based on Monte Carlo estimation.

¶ Continuous correction χ^2^ test.

### 3.2. Data cleaning

The threshold for data missing rate was set at 30%, and features with a missing rate exceeding 30% were eliminated. For features with a missing rate not exceeding 30%, MI was used to fill in the missing data. After data cleaning, 63 features remained for further analysis. The 1238 cases of data were then randomly divided according to a random seed of 42, with the training set and test set being split at an 8:2 ratio. The total number of samples in the training set was 990, and the total number of samples in the test set was 248.

### 3.3. Screening for risk factors of deep vein thrombosis in patients after total knee arthroplasty (TKA)

**Figure 1.**
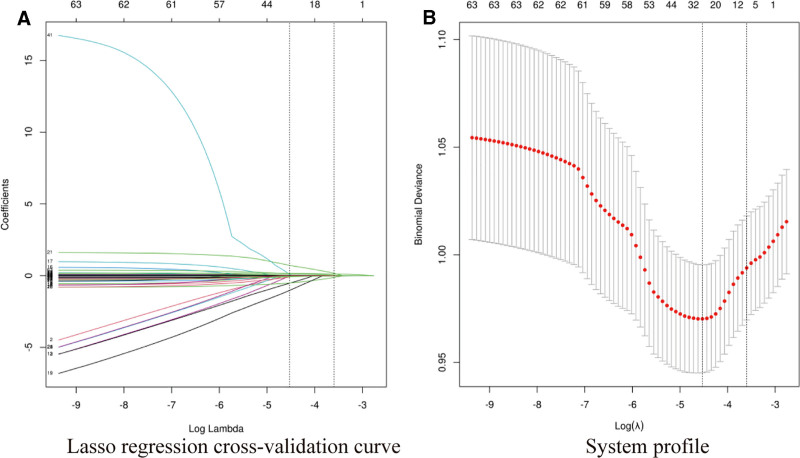
(A) Plot of the vertical line at the selected value using 10-fold cross validation, where the optimal lambda yields 11 nonzero coefficients. (B) The distribution of coefficients for 63 textural features is plotted from the log(λ) sequence in a LASSO model. The vertical dashed lines are drawn at the minimum mean squared error (λ = 0.011) and minimum distance standard error (λ = 0.027).

LASSO regression analysis was performed on the independent variables included in the study, with postoperative DVT as the dependent variable (Fig. 1). LASSO can compress variable coefficients to prevent overfitting and solve serious collinearity problems. The results showed that the minimum mean square error λ = 0.011, reducing 63 independent variables to 28, including history of diabetes, sleep apnea syndrome, myocardial infarction, allergy history, digestive system diseases, anemia, peripheral vascular disease, physical status, Ramsay sedation score, age, length of hospital stay, blood transfusion volume, blood loss, BMI, RCRI index, HCT, WBC, PLTS, APTT, D- dimer, AST, GLU, CL, Urea, TT, anesthesia time, operation time, postoperative movement analgesia score.

**Figure 2.**
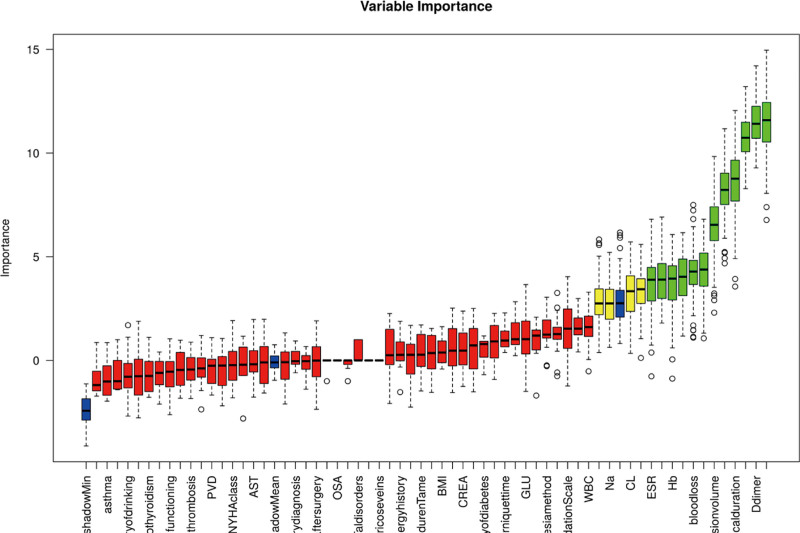
the Tentative (doubtful areas) are represented in yellow, Rejected (rejected areas) in red, Accepted (acceptable areas) in green, and Shadow (shadow features) in blue.

To further control the influence of confounding factors, Boruta feature selection analysis was applied to 63 independent variables. Among these, variables within the acceptable range include anemia, blood transfusion volume, blood loss, Hb, HCT, PT, FIB, D-dimer, TT, ESR, anesthesia time, and operation time. Variables in questionable areas include APTT, Na, CL, and postoperative pain score from physical activity. The selected variables are as shown in Figure 2, which includes anemia, blood transfusion volume, blood loss, Hb, HCT, PT, FIB, D-dimer, TT, ESR, anesthesia time, operation time, APTT, Na, CL, and postoperative pain score from physical activity.

Finally, the common variables screened out by the 2 methods were selected, namely anemia, blood transfusion volume, blood loss, HCT, D-dimer, TT, CL, anesthesia time, operation time, APTT, and postoperative movement pain score. These 11 independent variables were used as feature factors.

### 3.4. Modeling and performance comparison of multiple machine learning models

**Figure 3.**
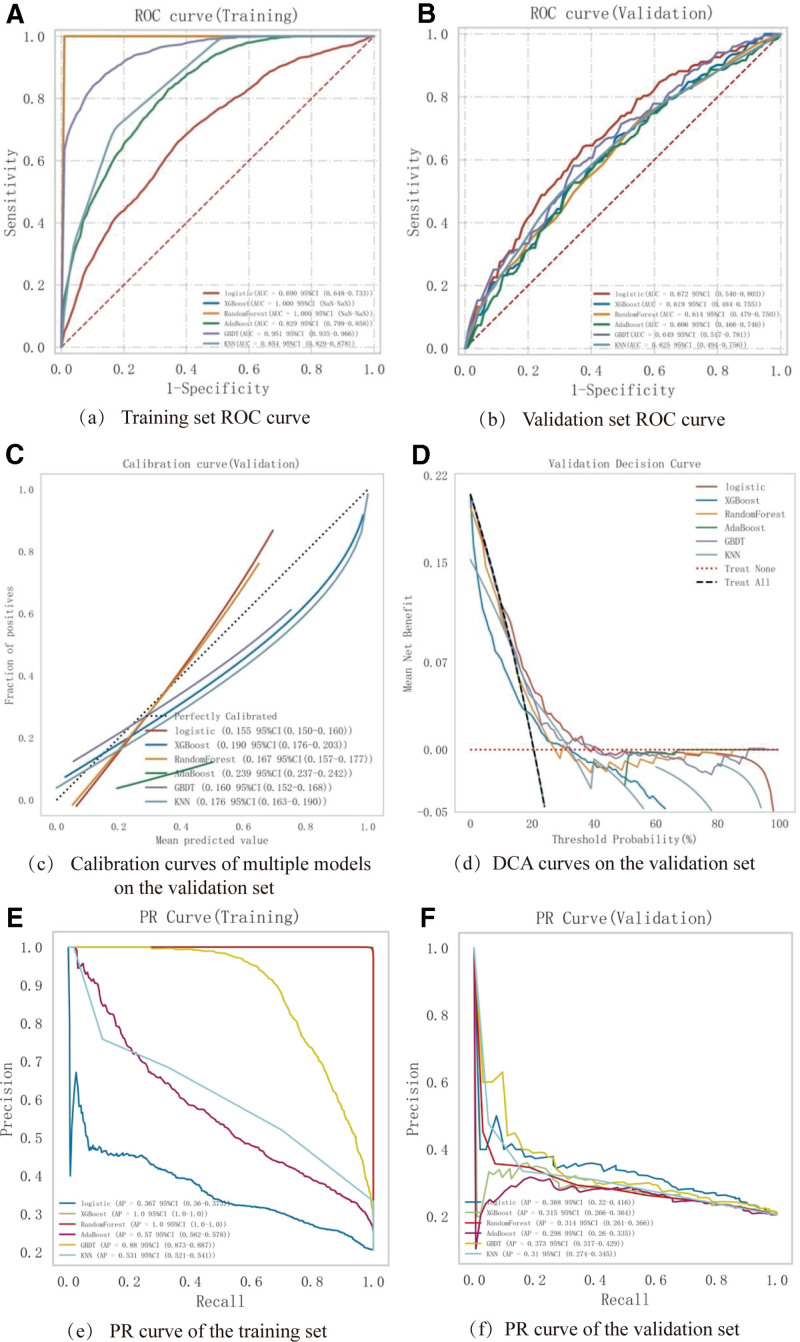
Comprehensive analysis of 6 machine learning models. (A) ROC curves and AUC values for training set and (B) ROC curves and AUC values for testing set. Patients after TKA were sampled 10 times at a ratio of 8:2. (C) Calibration curves for the testing set, where the abscissa is the average predicted probability, the case coordinate is the actual probability of the event, the dotted diagonal line is the reference line, and the other smooth solid lines are different model fitting lines. The closer the fitting line is to the reference line, the smaller the value in the brackets, and the more accurate the model prediction value. (D) Testing set DCA, where the black dashed line indicates the assumption that all patients have lower extremity DVT, and the red dashed line indicates the assumption that no patients have lower extremity DVT. The remaining solid lines indicate different models. (E) Training set PR curve and AP and (F) Testing set PR curve and AP. The y-axis is precision, and the x-axis is recall. If one model’s PR curve is completely covered by another model’s PR curve, it can be concluded that the latter is superior to the former, and the higher the AP value, the better the model performance. Different colors in the picture represent corresponding models, and values are represented by mean and 95% CI.

In the training set, predictive models for the occurrence of DVT in the lower limbs of patients after TKA were established using 6 machine learning algorithms: Logistic Regression (LR), eXtreme Gradient Boosting (XGBoost), Random Forest (RF), AdaBoost (Adaptive Boosting), Gradient Boosting Decision Tree (GBDT), and K- nearest neighbors (KNN), as shown in Table 2. A grid search method with 10-fold cross-validation was used to optimize the hyperparameters of the machine learning models. Finally, the performance of the 6 machine learning models was comprehensively evaluated and compared using the receiver operating characteristic curve - area under curve (ROC- AUC), cutoff value, accuracy, sensitivity, specificity, positive predictive value, negative predictive value, F1 score, and Kappa value (Table 3), and the ROC curves for the training set and validation set are shown in Figure 3A and 3B, respectively. According to the AUC values, the results showed that the XGBoost model and the Random Forest model had the highest values in the training set, while the Logistic model had the highest value in the test set. The AUC indicator focuses on the predictive accuracy of the model and does not indicate whether the model has clinical applicability, or which one is preferable. Therefore, DCA, calibration curves, and PR curves were also analyzed. The calibration curves showed that the GBDT model and the Logistic model had higher prediction accuracy (Fig. 3C). The DCA evaluation indicated that the Logistic model and the Random Forest model had better clinical applicability (Fig. 3D). In both the training and test sets, the AP value of the Logistic model was relatively stable (Figure 3E, F). A comprehensive analysis suggests that Logistic can be considered the optimal model.

**Table 2:** Multi-model classification - training set results summary.

ML model	AUC (95% CI)	Cutoff (95% CI)	Accuracy (95% CI)	Sensitivity (95% CI)	Specificity (95% CI)	PPV (95% CI)	NPV (95% CI)	F1 score (95% CI)	Kappa(95%CI)
logistic	0.691 (0.648_–_0.733)	0.194 (0.188–0.200)0.76	0.62 (0.603–0.637)	0.693 (0.669–0.717)	0.601 (0.574–0.628)	0.31 (0.303–0.318)	0.884 (0.879–0.888)	0.428 (0.423–0.432)	0.202 (0.192–0.212)
XGBo ost	1.000 (NaN-NaN)	6 (0.757–0.775)0.43	0.999 (0.999–0.999)	0.995 (0.995–0.995)	1.0 (1.000–1.000)	1.0 (1.000–1.000)	0.999 (0.999–0.999)	0.997 (0.997–0.997)	0.997 (0.997–0.997)
RandomFores t	1.000 (NaN-NaN)	5 (0.420–0.45	0.998 (0.998–0.999)	0.992 (0.989–0.996)	1.0 (0.999–1.000)	0.999 (0.997–1.000)	0.998 (0.997–0.999)	0.996 (0.994–0.997)	0.994 (0.992–0.997)
AdaBo ost	0.829 (0.799_–_0.858)	0)0.492 (0.492–0.493)	0.708 (0.694–0.722)	0.813 (0.790–0.836)	0.681 (0.658–0.703)	0.398 (0.385–0.410)	0.934 (0.929–0.940)	0.533 (0.526–0.540)	0.356 (0.342–0.369)
GBDT	0.951 (0.935_–_0.967)	0.239 (0.225–0.253)	0.88 (0.864–0.897)	0.855 (0.830–0.880)	0.887 (0.860–0.914)	0.67 (0.631–0.709)	0.96 (0.954–0.966)	0.748 (0.729–0.767)	0.672 (0.643–0.701)
KNN	0.854 (0.829–0.878)	0.4 (0.400–0.400)	0.831 (0.827–0.835)	0.329 (0.315–0.343)	0.961 (0.958–0.964)	0.684 (0.665–0.703)	0.847 (0.844–0.850)	0.444 (0.429–0.459)	0.359 (0.343–0.375)

CI = confidence interval, GBDT = gradient boosting decision tree, KNN = K-nearest neighbors, NPV = negative predictive value, PPV = positive predictive value, XGBoost = eXtreme gradient boosting.

**Table 3:** Multi-model classification - validation set results summary.

ML model	AUC (95% CI)	Cutoff (95% CI)	Accuracy (95% CI)	Sensitivity (95% CI)	Specificity (95%CI)	PPV (95% C I)	NPV (95% CI)	F1 score (95% CI)	Kappa (95% CI)
logistic	0.672 (0.541–0.804)	0.194 (0.188–0.200)	0.609 (0.591–0.627)	0.641 (0.582–0.700)	0.601 (0.566–0.636)	0.293 (0.283–0.303)	0.868 (0.854–0.882)	0.4 (0.384–0.417)	0.167 (0.148–0.186)
XGBoo st	0.620 (0.484–0.756)	0.766 (0.757–0.775)	0.779 (0.766–0.791)	0.049 (0.021–0.077)	0.967 (0.952–0.982)	nan(NaN-NaN)	0.798 (0.792–0.803)	nan(NaN-NaN)	0.024 (-0.025–0.073)
Random Forest	0.621 (0.485–0.757)	0.435 (0.420–0.450)	0.784 (0.762–0.806)	0.178 (0.105–0.250)	0.94 (0.925–0.956)	0.428 (0.288–0.568)	0.816 (0.801–0.831)	0.245 (0.156–0.334)	0.147 (0.053–0.241)
AdaBoo st	0.606 (0.466–0.746)	0.492 (0.492–0.493)	0.602 (0.565–0.639)	0.538 (0.473–0.603)	0.619 (0.572–0.666)	0.269 (0.239–0.299)	0.838 (0.817–0.859)	0.357 (0.319–0.394)	0.115 (0.061–0.170)
GBDT	0.645 (0.515–0.776)	0.239 (0.225–0.253)	0.678 (0.650–0.705)	0.429 (0.353–0.506)	0.742 (0.707–0.777)	0.298 (0.253–0.344)	0.835 (0.818–0.852)	0.349 (0.293–0.405)	0.146 (0.080–0.212)
KNN	0.625 (0.494–0.756)	0.4 (0.400–0.400)	0.766 (0.741–0.790)	0.178 (0.118–0.239)	0.917 (0.897–0.938)	0.351 (0.233–0.469)	0.812 (0.798–0.827)	nan(NaN-NaN)	0.117 (0.030–0.203)

CI = confidence interval, GBDT = gradient boosting decision tree, KNN = K-nearest neighbors, NPV = negative predictive value, PPV = positive predictive value, XGBoost = eXtreme gradient boosting.

### 3.5. Optimal model construction and evaluation

**Figure 4.**
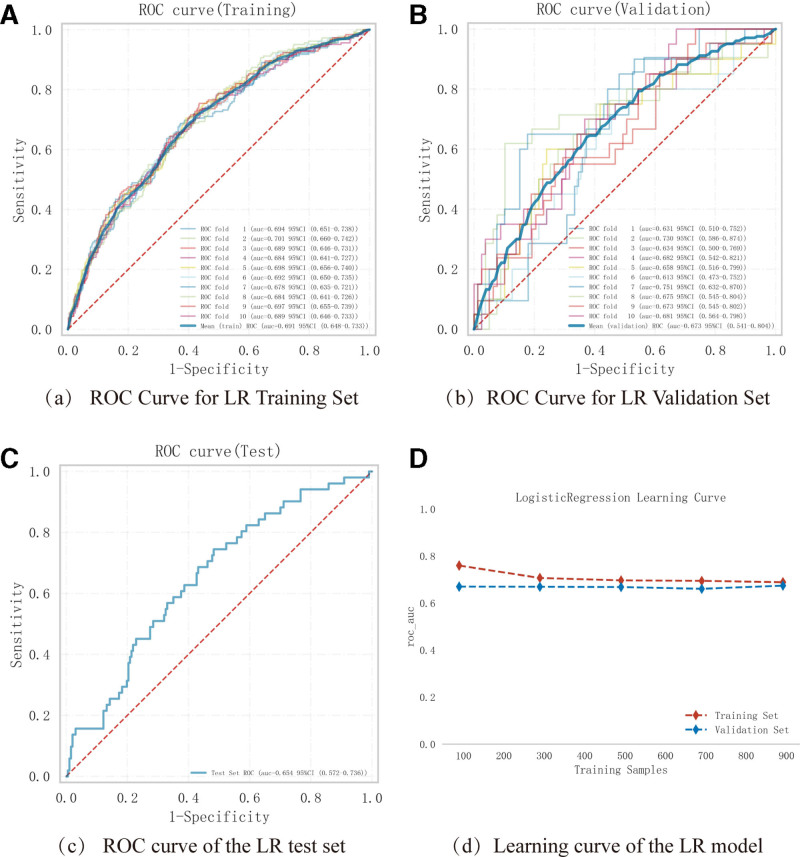
ROC values and learning curves of the training set, validation set, and test set for the logistic regression model. (A) The ROC curve and AUC value of the training set and (B) the ROC curve and AUC value of the validation set after ten-fold cross-validation, with different colors of solid lines representing 10 different results. (C) The ROC curve and AUC value of the test set. (D) Learning curves. The red dashed line represents the training set, and the blue dashed line represents the validation set. These values are presented as mean and 95% CI.

Logistic regression analysis and 10-fold cross-validation were performed on the training set. The results showed that the average AUC of the training set was 0.691 (0.648–0.733), the average AUC of the validation set was 0.673 (0.541–0.804), and the AUC of the test set was 0.654 (0.572–0.736) (Fig. 4A–C). TheAUCs ofthe training, validation, and test sets eventually stabilized at around 0.673, indicating accurate model prediction performance. Given that the performance under the AUC metric for the validation set is lower than that of the test set or exceeds <10%, it can be considered that the model fitting is successful, and the learning curve indicates strong fitting and high stability for both the training and validation sets (Fig. 4D). These results suggest that the Logistic model can be used for classification modeling tasks with this dataset.

### 3.6. SHAP value interpretation model

**Figure 5.**
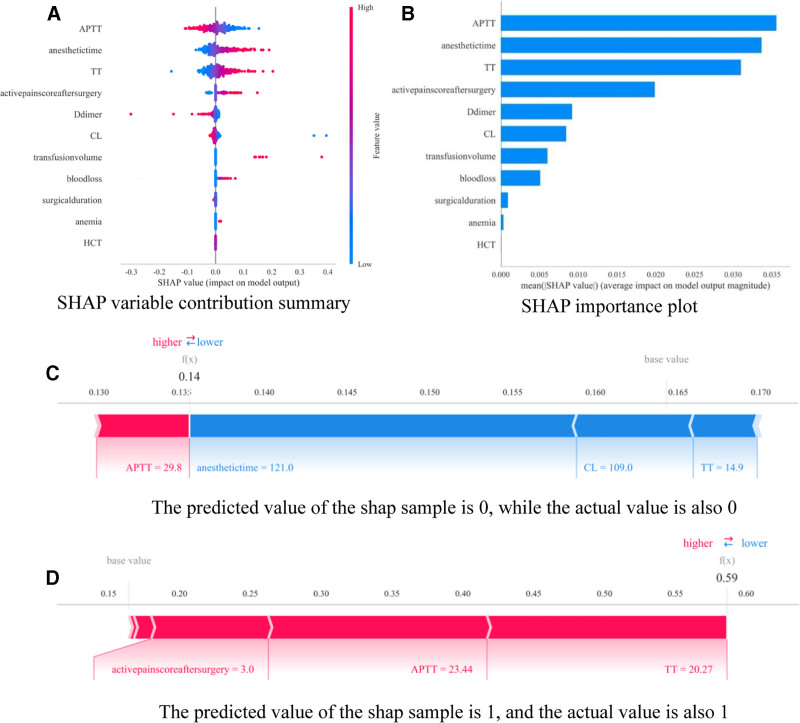
SHAP explanation model. (A) Feature attributes in SHAP. Each line represents a feature, and the horizontal axis is the SHAP value. The red points represent higher feature values, and the blue points represent lower feature values. (B) Ranking of feature importance by SHAP value. The matrix plot describes the importance of each covariate in the final prediction model development. (C) Individual predicted values for patients without DVT and (D) individual predicted values for patients with DVT. The SHAP value indicates the predictive features of individual patients and the contribution of each feature to predicting an adverse outcome. The bold number is the probability predicted value (f(x)), while the base value is the predicted value without input to the model. f(x) is the log-odds ratio for each observation. Red features indicate increased risk, and blue features indicate decreased risk. The length of the arrow helps visualize how much the prediction is affected. The longer the arrow, the better the effect.

To visually interpret the selected variables, we used SHAP values to illustrate how these variables predicted the formation of lower extremity DVT in patients after TKA. Figure 5A shows the 11 most important features in our model. In each feature importance line, the attribution of all patients to the outcome is plotted with different color points, where red points indicate high-risk values and blue points indicate low-risk values. APTT shortening, longer anesthesia time, elevated TT, higher postoperative motor analgesia score, decreased D-dimer, decreased CL, increased blood transfusion volume, increased blood loss, shorter operation time, anemia and will increase the risk of deep vein thrombosis in the lower extremities of patients after TKA. Figure 5B shows the ranking of 11 risk factors evaluated by the average absolute SHAP value, where the x-axis SHAP value indicates the importance of the prediction model, among which APTT has the highest importance. In addition, we provided 2 typical examples to illustrate the interpretability of the model, one was a patient with gout without gout, and the SHAP prediction score was low (0.14) (Fig. 5C), while another patient with gout had a higher SHAP score (0.59) (Fig. 5D).

## 4. Discussion

The purpose of this study is to construct a predictive model for the risk of DVT in the lower extremities of patients after TKA using machine learning methods, and to analyze the interpretability of the model using the SHAP method. We used the clinical data of 1238 patients who underwent TKA at the Second Hospital of Shanxi Medical University. After data preprocessing and cleaning, 11 clinical feature variables were selected from 63 clinical variables through LASSO regression and Boruta feature screening, namely anemia, blood transfusion volume, blood loss, HCT, D-dimer, TT, CL, anesthesia time, operation time, APTT, postoperative exercise analgesia score, to assess the risk of lower extremity DVT in patients after TKA. Six machine learning models were selected and established, and the models were compared and evaluated by various evaluation indicators such as AUC value, cutoff value, accuracy, sensitivity, specificity, positive predictive value, negative predictive value, F1 score, and Kappa value. It was found that after analyzing AUC, DCA, calibration curve and PR curve, the logistic regression model performed better than the other 5 machine learning models. However, to more comprehensively interpret the machine learning prediction model and intuitively present the prediction results to clinicians, we applied the SHAP method to the logistic regression model to achieve the best prediction effect and interpretability. The results showed that factors such as APTT, anesthesia time, TT, postoperative exercise analgesia score, D-dimer are important features affecting the risk of DVT in patients after TKA.

In this study, we found clinical factors that may affect the formation of DVT after TKA, such as shortened APTT, longer anesthesia time, elevated TT, higher postoperative motor analgesia score, decreased D-dimer, decreased CL, increased blood transfusion volume, increased blood loss, shorter operation time and anemia. Among them, APTT was the most important predictor. APTT is a laboratory test for evaluating the function of the intrinsic coagulation system, and shortened APTT usually means that the blood is in a hypercoagulable state, so it is an independent risk factor for thrombosis.^[4]^ The increase of anesthesia time may lead to blood stasis, activation of coagulation function, endothelial injury, hemodynamic changes, etc, which increase the risk of thrombosis. TT is a test index of blood coagulation function, and its elevation may indicate abnormal coagulation function, thus increasing the risk of thrombosis, so TT elevation is a risk factor for thrombosis. The relationship between elevated postoperative motor analgesia score and thrombosis is not direct, but the elevated score may indirectly reflect the poor recovery of patients after surgery, thus indirectly increasing the risk of thrombosis. Studies have shown that decreased D-dimer is a predictor, which is different from previous studies, probably because only preoperative data were collected, however, since most of the patients are elderly people with other complications, they may have been taking anticoagulants for a long time, or have undergone preoperative measures such as air pressure, ankle pump, limb activity, muscle contraction and other thrombosis prevention measures, leading to decreased D-dimer, which is also a shortcoming of this study. In addition to collecting preoperative laboratory data, preoperative and postoperative clinical measures and postoperative laboratory data should also be collected to make the research results more convincing. When CL decreases, the concentration of acetylcholine at the nerve endings increases, which may lead to increased neural excitability, thereby increasing the risk of thrombosis. Increased blood transfusion volume may lead to increased blood viscosity, thus increasing the risk of thrombosis. In addition, blood transfusion itself may also have an impact on the blood coagulation system. For example, infusion of a large amount of red cell suspension may lead to platelet aggregation and activation of coagulation factors, thus increasing the risk of thrombosis.^[5]^ Blood loss will lead to a decrease in the number of red blood cells in the blood, which will make the blood more viscous. In addition, blood loss will also lead to a decrease in the number of coagulation factors and platelets in the blood, which will also increase the risk of forming thrombosis. On the other hand, thrombosis itself may also lead to blood loss. When thrombosis forms inside the blood vessels, it may block the blood vessels and cause local ischemia. If the ischemia is severe enough, it will lead to tissue necrosis and bleeding. Therefore, increased blood loss is one of the risk factors for thrombosis. The shortening of operation time may lead to a decrease in postoperative bed rest time and an increase in patient activity, which may increase the risk of thrombosis. In addition, thrombosis itself may also lead to a shortening of operation time. Anemia is a disease characterized by insufficient quantity or quality of red blood cells in the blood, leading to inadequate oxygen supply. When the body is in an anemic state, it may increase the risk of thrombosis. According to previous studies, a study in 2014 used the global thrombus test (GTT) to examine the thrombotic and fibrinolytic status of non-anticoagulated blood to develop markers for predicting the incidence of DVT after TKA.^[6]^ The study found that preoperative GTT-lysis time (LT), an indicator of thrombotic activity, was significantly shorter in the DVT group than in the non-DVT group. This suggests that enhanced preoperative thrombotic activity assessed by GTT may be a predictive marker for the occurrence of DVT after TKA. In 2018, Wanli et al evaluated the relationship between preoperative serum leptin levels and the incidence of postoperative venous thromboembolism in patients with osteoarthritis (OA) undergoing total knee arthroplasty in their hospital.^[7]^ The results showed that high preoperative leptin levels may be an independent risk factor for postoperative DVT. In the same year, another study analyzed the thrombotic occurrence in 146 patients undergoing total knee arthroplasty to explore the risk factors and coping strategies for postoperative lower extremity DVT.^[8]^ The study results showed that advanced age, elevated D-dimer, decreased prothrombin time international normalized ratio (PT-INR), high body mass index, and comorbid internal diseases are risk factors for DVT after TKA. Finally, 2 studies in 2023 analyzed the potential risk factors for postoperative DVT in patients over 60 years old undergoing TKA surgery^[9]^ and the risk factors for preoperative DVT in patients with knee OA undergoing TKA.^[10]^ Among them, the first study found that preoperative HCT, platelet count, anesthesia method, and diabetes were independent risk factors; the second study analyzed the risk factors for preoperative DVT. Overall, the following contributions were made in this study. Firstly, a highly efficient and interpretable machine learning model was developed to predict the risk of DVT in patients after TKA, which helps in clinical identification of high-risk patients and providing personalized interventions, reducing the risk of DVT after TKA and improving patient prognosis. The Logistic model established in this study has a high sensitivity and specificity and can be used as an auxiliary diagnostic tool in clinical applications, helping medical staff to timely identify and treat DVT patients after TKA, thereby reducing serious complications of DVT, such as pulmonary embolism and pulmonary heart disease, and improving patient quality of life and survival period. Secondly, it revealed the key clinical features that affect the occurrence of DVT after TKA and their degree of influence, which helps to deepen the understanding of the mechanism of DVT occurrence and provide a basis for formulating more reasonable and effective prevention strategies. These findings are not only consistent with existing literature reports, but also provide new clues and directions for further exploration of the mechanism of DVT occurrence. Based on these findings, more reasonable and effective prevention strategies can be formulated for different risk factors in clinical practice, such as providing stronger or longer-term anticoagulation treatment and lifestyle adjustments for patients with shortened APTT; to avoid prolonged anesthesia time, assess patient condition and adjust anesthetic dosage before anesthesia, doctors and anesthetists work closely together to ensure the consistency of surgical plans and anesthesia plans; for patients with high postoperative exercise pain scores, physicians can adjust the type or dosage of analgesic drugs according to the severity of pain, adopt multimodal analgesia strategies and physical and psychological treatments. Secondly, it explored a new approach to interpretability analysis of machine learning models using the SHAP method, which helps to enhance the transparency and credibility of machine learning models in the medical field and provides more intuitive and reliable decision support for medical staff. This study applied the SHAP method to the machine learning model for predicting the risk of DVT in patients after TKA, which can quantify the contribution of each feature to the prediction results, thus helping medical staff understand how the model makes predictions and recommendations based on patient features, and which features have a significant impact on the prediction results. This not only enhances the trust and acceptance of medical staff in the model, but also provides more intuitive and reliable decision support for medical staff.

However, our study has several limitations. Firstly, the research data is derived from a single-center retrospective study, not a multi-center study, which may have selection bias, affecting the representativeness and generalizability of the research results, so the universality of the results is limited. Secondly, although high consistency was achieved in the reproducibility analysis in the training and testing sets, some inevitable errors may occur due to segmentation uncertainty. In addition, the research data we collected only contains some clinical variables, possibly overlooking some important factors related to DVT risk, such as genetic factors, drug factors, clinical measures, lifestyle factors, etc, and some data only collected preoperative data, missing postoperative data, affecting the integrity and depth of the research results.

Longitudinal or prospective case-control studies are also needed to further explain the relationship between risk factors and DVT formation.

In summary, APTT, anesthesia duration, TT, postoperative motor analgesia score, and D-dimer are high-risk factors for DVT after TKA. The Logistic model established in this study has relatively stable performance and can be used as an auxiliary diagnostic tool in clinical applications to help medical staff timely identify and treat DVT after TKA. However, more cases and factors related to the risk of DVT occurrence need to be collected in the future, and multicenter or cross-regional validation is required. In conclusion, through this model, high-risk groups for DVT after TKA can be identified, and strengthening the management of these groups may help reduce the incidence of DVT after TKA.

## Author contributions

**Conceptualization:** Huanya Li, Zhicheng He, Li Guo.

**Data curation:** Huanya Li, Zhicheng He.

**Formal analysis:** Huanya Li.

**Investigation:** Huanya Li.

**Methodology:** Huanya Li, Li Guo.

**Project administration:** Xiaochun Wei.

**Resources:** Pengcui Li, Xiaochun Wei.

**Supervision:** Pengcui Li, Li Guo, Xiaochun Wei.

**Validation:** Huanya Li, Zhicheng He, Pengcui Li, Li Guo, Xiaochun Wei.

**Writing – original draft:** Huanya Li.

**Writing – review & editing:** Huanya Li, Zhicheng He.
